# Regionally Specific White Matter Disruptions of Fornix and Cingulum in Schizophrenia

**DOI:** 10.1371/journal.pone.0018652

**Published:** 2011-04-14

**Authors:** Muhammad Farid Abdul-Rahman, Anqi Qiu, Kang Sim

**Affiliations:** 1 Division of Bioengineering, National University of Singapore, Singapore, Singapore; 2 Clinical Imaging Research Center, National University of Singapore, Singapore, Singapore; 3 Singapore Institute for Clinical Sciences, the Agency for Science, Technology and Research, Singapore, Singapore; 4 Research Department, Institute of Mental Health, Singapore, Singapore; 5 Department of General Psychiatry, Institute of Mental Health, Singapore, Singapore; University of California, San Francisco, United States of America

## Abstract

Limbic circuitry disruptions have been implicated in the psychopathology and cognitive deficits of schizophrenia, which may involve white matter disruptions of the major tracts of the limbic system, including the fornix and the cingulum. Our study aimed to investigate regionally specific abnormalities of the fornix and cingulum in schizophrenia using diffusion tensor imaging (DTI). We determined the fractional anisotropy (FA), radial diffusivity (RD), and axial diffusivity (AD) profiles along the fornix and cingulum tracts using a fibertracking technique and a brain mapping algorithm, the large deformation diffeomorphic metric mapping (LDDMM), in the DTI scans of 33 patients with schizophrenia and 31 age-, gender-, and handedness-matched healthy controls. We found that patients with schizophrenia showed reduction in FA and increase in RD in bilateral fornix, and increase in RD in left anterior cingulum when compared to healthy controls. In addition, tract-based analysis revealed specific loci of these white matter differences in schizophrenia, that is, FA reductions and AD and RD increases occur in the region of the left fornix further from the hippocampus, FA reductions and RD increases occur in the rostral portion of the left anterior cingulum, and RD and AD increases occur in the anterior segment of the left middle cingulum. In patients with schizophrenia, decreased FA in the specific loci of the left fornix and increased AD in the right cingulum adjoining the hippocampus correlated with greater severity of psychotic symptoms. These findings support precise disruptions of limbic-cortical integrity in schizophrenia and disruption of these structural networks may contribute towards the neural basis underlying the syndrome of schizophrenia and clinical symptomatology.

## Introduction

Limbic circuitry disruptions have been implicated in the psychopathology and cognitive deficits of schizophrenia [1], [2], [3]. The fornix and cingulum bundles are the most prominent white matter fiber tracts within the limbic system. The fornix is a major white matter bundle projection from the hippocampus to other brain structures, including the mamillary bodies, thalamus, septal region, nucleus accumbens, whereas the cingulum connects the cingulate cortex with the prefrontal cortex, premotor regions, cortical association areas in the parietal and occipital lobes, parahippocampal cortex and the thalamus [4], [5]. With regard to the cingulate gyrus, earlier post-mortem studies have observed altered neuronal arrangement [6], smaller pyramidal cells [7] and reductions in the oligodendrocytes within this brain region [8]. Structural MRI studies have reported gray matter abnormalities in the cingulate gyrus, especially in the anterior segment in schizophrenia [9], [10], [11], [12]. With respect to the fornix, previous neuropathological studies have found no difference in fornix volume [13], but increased fiber density of the left fornix in male patients with schizophrenia [14]. Looking at early onset cases, Davies et al [15] found an increase in the cross sectional area of the fornix in his study of subjects with schizophrenia. Taken together, these data suggest that structural disruptions of the fornix and cingulum bundles may occur in schizophrenia.

Diffusion tensor imaging (DTI) is a specific neuroimaging technique that enables measure of the restricted water diffusion in brain tissue. Fractional anisotropy (FA) derived from the diffusion tensor may be influenced by myelination, orientation, coherence, packing density and structural integrity of neural fiber tracts [16], [17]. Hence, reduction in FA often points to possible structural abnormalities in neural fiber tracts. To better understand biological processes behind changes in FA, radial diffusivity (RD) and axial diffusivity (AD) derived from the diffusion tensor model can be utilized to predict pathophysiological disruptions such as demyelination [18], [19] or axonal damage [20].

Voxel or region of interest [21] based assessments of the cingulum and fornix bundles have been employed to study FA alterations in schizophrenia [1], [22], [23], [24], [25], primarily pursuing the hypothesis that schizophrenia would be associated with FA differences in these two bundles. Reductions of FA in the cingulum have been found in some [21], [26], [27] but not all DTI studies of patients with schizophrenia [28], [29]. Extant examinations of the fornix using DTI have found reductions of FA in schizophrenia [1], [25], [30] but Kendi et al [31] specifically investigated this limbic bundle and did not find differences in FA in their study of early onset patients with schizophrenia. The observed discrepancies between studies may be due to clinical differences in the populations studied as well as methodological differences in anatomical definitions of these two bundles. Misalignment of anatomy in voxel-based analysis and segmentation errors of white matter bundles in ROI-based analysis may exaggerate partial volume effects. As consequences, they may increase random effects on FA and thus decrease statistical power in detecting abnormalities of the fornix and cingulum regions in schizophrenia. One potential way to resolve this is to reliably delineate the core of these fiber bundles and to localize FA abnormalities along the entire white matter bundle.

To date, efforts to examine the white matter differences within the limbic white matter bundles (cingulum and fornix) in a detailed manner covering the entire tracts have been sparse. Recently, Segal et al [32] manually traced the anterior and posterior cingulate gyri, divided the anterior cingulate gyrus axially into six equal segments, and the posterior cingulate gyrus into two segments in their quest to understand the regional differences within the cingulate gyrus. Unlike earlier approaches [23], [24], [25] that have largely limited FA quantification to averaged values within the ROIs of these fiber tracts, we mapped FA, RD, and AD values along the entire fiber bundles of the fornix and cingulum using a tractographic approach [33] and with geometric representation of these bundles generated using large deformation diffeomorphic metric mapping (LDDMM) [34] in this study. In particular, we employed fibertracking on an averaged DTI over the subjects in our study such that the fornix and cingulum were delineated only in the region that is most overlapped across our samples. This potentially reduces partial volume effects on statistical testing of the diffusion measurements. Additionally, our method facilitated the detection of FA, RD, and AD differences with respect to the structural geometry of each fiber bundle, which can potentially clarify regionally specific white matter abnormalities within the fornix and cingulum in schizophrenia.

## Materials and Methods

### 2.1 Subjects

Thirty three right-handed patients with schizophrenia and thirty one age-, gender- and handedness-matched healthy controls were recruited from the Institute of Mental Health, Singapore and the community respectively for this study. The study was approved by the Institutional Review Boards of the Institute of Mental Health, Singapore, as well as that of the National Neuroscience Institute, Singapore. All subjects gave their written informed consent following a complete description of the study. Socio-demographic and clinical information for the two groups of subjects are given in Table 1.

**Table 1:** Demographic and clinical characteristics of the sample.

Demographic/clinical feature	CON(N = 31)	SCZ(N = 33)	Test statistic	P value
Age (SD), years	35.4 (8.82)	39.4 (8.82)	*t_62_* = −1.844	0.0744
Sex (% Male)	77.4% (24/31)	76.0% (25/33)	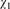 = 2.35	0.8754
Years of Education (SD), years	13.9 (2.50)	11.6 (2.40)	*t_62_* = 3.700	0.0005
Mean Illness Duration (SD), years	-	12.7 (8.99)	-	-
PANSS positive symptom subscale	-	10.1(3.28)	-	-
PANSS negative symptom subscale	-	8.7(2.68)	-	-
PANSS general psychopathology subscale	-	19.9 (4.17)	-	-
GAF scores	-	53.6 (17.5)	-	-

Abbreviations: CON, control subjects; SCZ, patients with schizophrenia; SD, standard deviation.

All diagnoses were made by a psychiatrist using information obtained from the clinical history, mental status examination, existing medical records, interviews with significant others as well as the administration of the Structured Clinical Interview for DSM-IV disorders-Patient Version (SCID-P) [35]. The patients were maintained on a stable dose of antipsychotic medications for at least two weeks prior to recruitment and did not have their medications withdrawn for the purpose of the study. Twenty five patients received second generation antipsychotics, seven patients were prescribed first generation antipsychotics and one patient was on a combination of first and second generation antipsychotics. The mean (SD) antipsychotic dose was 200.15 (153.73) daily chlorpromazine equivalents in milligrams. No subject met DSM-IV criteria for alcohol within the three months prior to the scan or other lifetime substance abuse. The Positive and Negative Syndrome Scale (PANSS) [36] was used to assess the nature and severity of psychopathology, while the Global Assessment of Functioning Scale [37] was used to assess the level of psychosocial functioning. Both scales were administered by a psychiatrist to all the participants. The healthy controls were screened using the SCID-NP [38] to be free of any Axis I psychiatric disorder. None of the subjects had a history of major neurological, medical illnesses, substance abuse or psychotropic medication use.

### 2.2 DTI acquisition and preprocessing

Single-shot echo-planar DTI (TR = 3725 ms; TE = 56 ms; flip angle = 90°) was acquired using a 3-Tesla whole body Philips scanner with a SENSE head coil. 42 axial slices with 3.0 mm thickness were acquired parallel to the anterior–posterior commissure line; the imaging matrix was 112×109 with a field of view of 230 mm×230 mm, which was zero-filled to 256×256. 15 diffusion weighted images (DWIs) with b = 800 sec/mm^2^ and 1 baseline with b = 0 sec/mm^2^ were obtained.

Within each subject, DWIs were first corrected for motion and eddy current distortions using affine transformation to the image without diffusion weighting. These DWIs were then registered to Mori's single-subject DTI atlas (resolution: 1×1×1 mm^3^, http://www.mristudio.org) [5] via affine transformation between the images without diffusion weighting. To align subjects' diffusion tensor images to Mori's atlas, we employed the LDDMM image mapping to simultaneously align the subject's FA map and the image without diffusion weighting to those of Mori's single-subject DTI atlas [39]. We followed the same mapping procedure and LDDMM parameter setting as those given in [39]. The accuracy of this nonlinear registration approach has been extensively validated and reported elsewhere [39]. For each individual subject, the tensor, FA, RD, and AD maps were recomputed using the DWIs aligned to Mori's DTI atlas via the diffeomorphic transformation. In addition, the mean DWIs were also constructed by averaging the corresponding DWIs across healthy controls. The tensor and FA map were computed from the mean DWIs in Mori's atlas space to represent the white matter anatomy in this study.

### 2.3 Fornix and Cingulum Fiber Tracking

The fornix and cingulum bundles in each hemisphere were reconstructed from the mean DTI using the Fiber Assignment Continuous Tracking (FACT) method [33] with a FA threshold of 0.15 and an angle threshold of 50°. The FACT tracking was first performed from all pixels inside the brain. Then, each bundle of interest was extracted using a multi-ROI approach, that is, all tracts penetrating these ROIs were assigned to this bundle for further analysis. The protocol that defines these ROIs in the mean DTI color map, which is in Mori's DTI atlas space, for the fornix and cingulum bundles is elaborated below. The ROI drawing was repeatable given their coordinates in the Mori's DTI atlas space.

#### Fornix

**Figure 1:**
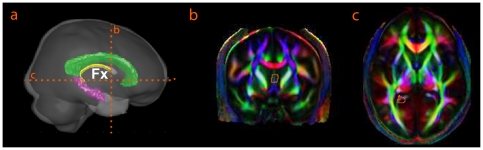
Delineation of the fornix bundle (Fx). Panel (a) illustrates the 3D view of the brain, where the fornix, hippocampus, corpus callosum are colored in yellow, pink, and green, respectively. Panels (b, c) show the coronal and axial slices of the mean DTI color map, where the ROIs are defined for delineating the fornix.

Two ROIs were manually drawn on the color map. The first ROI was placed on the most anterior coronal plane where the body of fornix is visible on the color map (**Figure 1**(b), coronal slice 150). Then, the second ROI was drawn on the axial plane that includes the tail of the hippocampus (**Figure 1**(c), axial slice 108). After the fornix bundle was extracted, the average values of FA, AD, and RD were calculated for the entire bundle, separately for each hemisphere.

#### Cingulum

**Figure 2:**
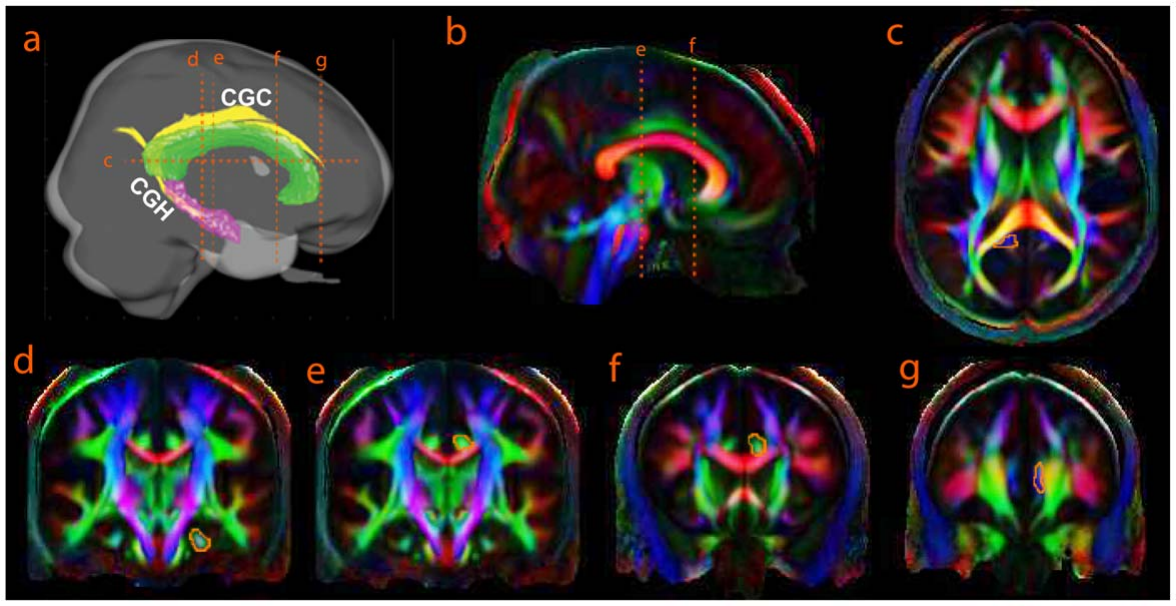
Delineation of the cingulum bundles. They include the segment adjoining the hippocampus (CGH) and the main cingulum (CGC). Panel (a) illustrates the 3D view of the brain, where the cingulum, hippocampus, and corpus callosum are colored in yellow, pink, and green. Panels (b–g) respectively show the sagittal, axial, and coronal slices of the average DTI color map, where the ROIs are defined for delineating CGH and CGC. CGH is defined by the ROIs given in panels (c, d) whereas CGC is defined by the ROIs given in panels (c, g). CGC is further divided into three partitions using the ROIs in panels (b, e, f).

The cingulum bundle can be divided into two segments, namely, the upper part along the main cingulate gyrus (CGC: cingulum in the cingulate gyrus) and the lower segment along the ventral side of the hippocampus (CGH: cingulum adjoining the hippocampus) [40]. Two ROIs were used to extract each segment of the cingulum bundle. For CGH, it runs along the ventral aspect of the hippocampus. The first ROI was defined on the axial plane where the cingulum bundle begins to turn anteriorly above the splenium (**Figure 2**(c), axial slice 126) and the second ROI was chosen on the coronal plane anterior to the pons (**Figure 2**(d), coronal slice 125). For CGC, the first ROI was defined on the axial plane where CGH ends (**Figure 2**(c), axial slice 126) and the second ROI was defined on the coronal slice where is the most anterior to the corpus callosum (**Figure 2**(g), coronal slice 183). CGC was further divided into three portions (anterior, middle, and posterior) based on the cortical projection of the corpus callosum. The two ROIs were respectively defined on the coronal planes (**Figure 2**(e, f), coronal slices 127 and 162) where the fiber orientation of the corpus callosum was changed (**Figure 2**(b)). After the four bundles were extracted, the average values of FA, RD and AD were calculated for each bundle, separately for each hemisphere.

#### Reliability

**Figure 3:**
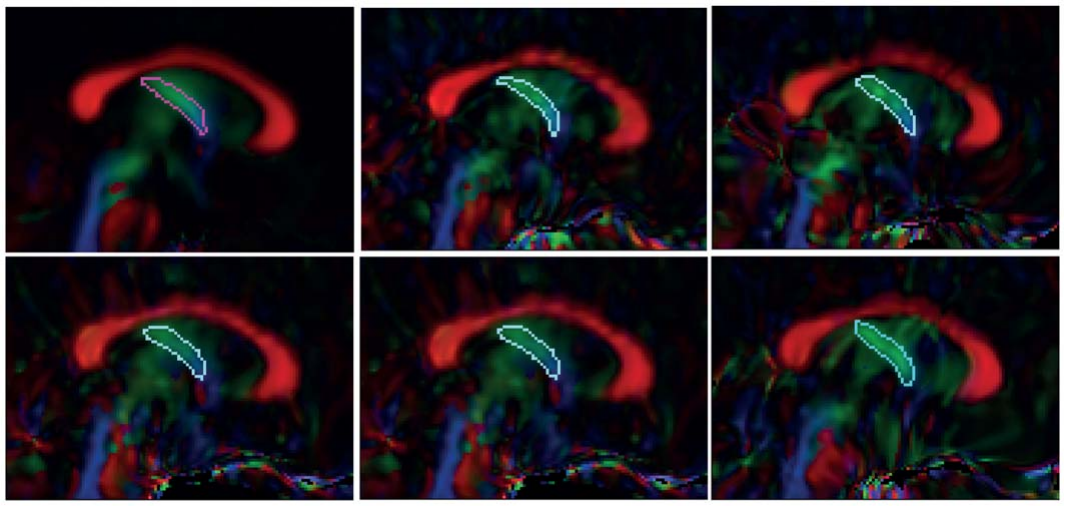
Fornix manual segmentation. Panel (a) shows the fornix mask extracted from the averaged DTI using the fiber tracking method. Panels (b–f) show the manual masks of the fornix labeled on the DTI of five subjects.

Both fornix and cingulum bundles were extracted using the FACT fibertracking approach on the averaged DTI. The coordinates of the multiple ROIs in Mori's DTI atlas where each bundle passes through were recorded and easily reproduced using the fibertracking method. However, since the tracking was performed only on the averaged DTI, misalignment of the DTI data across subjects could affect the result of the fiber tracking, especially for small structures like the fornix. To ensure that the fornix of each subject overlapped with the ROI of the fornix extracted from the averaged DTI using the fiber tracking method, we manually segmented the fornix of five subjects (see manual segmentation in **Figure 3**) and transformed the fornix mask to the atlas. The volume overlap ratio between the manual segmentation volume and the automatically extracted volume in Mori's atlas was computed. Its mean and standard deviation are 87.5% and 2.6%, indicating high reliability in the procedure of the fornix delineation within this study.

#### Mean Curve Generation

For each bundle, a mean curve was computed using the LDDMM curve mapping [34], [41] based on all the fiber tracts within this bundle. The mean curve was then parameterized at 30 points along its arc length and was used as the geometric representation of the bundle. The FA, RD and AD distributions along each bundle was characterized as a function of the arc length, and obtained for each subject. This allows the detection of local abnormalities at specific locations along each bundle of interest.

### 2.4 Statistical Analysis

Demographic variables between patients with schizophrenia and control subjects were compared using two sample Student's *t*-test for continuous variables and chi-square test for categorical variables.

### ROI-based Measures

Linear regression with the main effects of diagnostic group (control vs schizophrenia) and covariates of age, years of education, and illness duration were performed on the mean FA, RD and AD values in each bundle to elucidate abnormalities in schizophrenia.

### Tract-based Measures

To detect local differences for values of FA, RD and AD between the two diagnostic groups, the respective values at each point on the mean curve of each bundle was first smoothed by averaging the values of its closest five neighboring points along the curve. Linear regression with the main effects of diagnostic group (control versus schizophrenia) and covariates of age, years of education, and illness duration were then used to examine local differences in FA, RD and AD between the two diagnostic groups. The statistical results were visualized at an overall significance level of 0.05 after Bonferroni correction.

### Correlation with Clinical Measures

Partial correlation analysis was performed to investigate the relationship between the DTI measures within each ROI and psychopathological measures assessed by PANSS in patients with schizophrenia when controlling for age, years of education, and illness duration. To detect the specific location along each bundle where the DTI measure is associated with the psychopathology, the same partial correlation analysis was performed on every point of each bundle. The statistical results were visualized at an overall significance level of 0.05 after Bonferroni correction.

## Results

### Demographic and Clinical Characteristics

Compared to healthy controls, patients with schizophrenia had fewer years of education (p = 0.0005). There was no statistically significant difference in age (p = 0.0744) and sex (p = 0.8754) between the two groups. The average duration of illness in patients with schizophrenia was 12.7 years.

### ROI-based Findings

**Figure 4:**
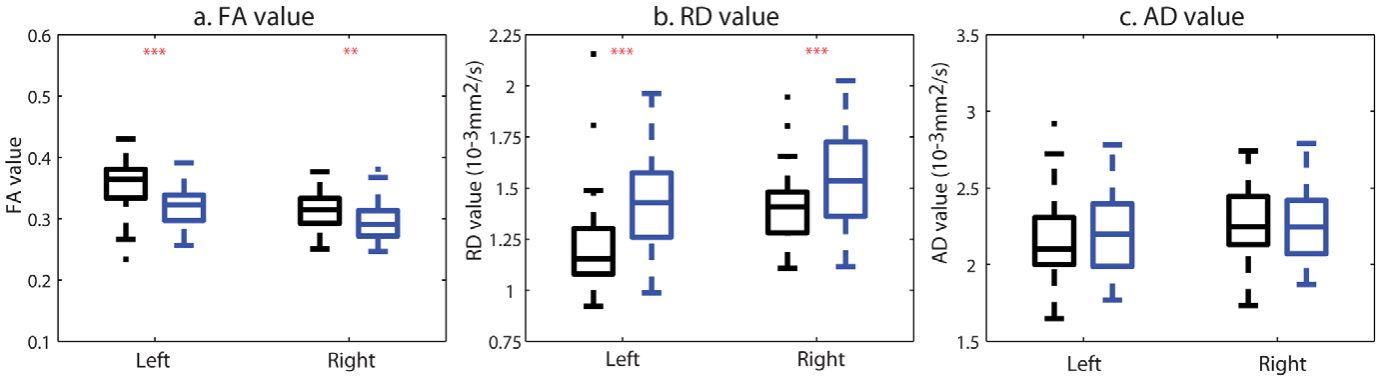
ROI-based analysis of the fornix DTI measures. Panels (a, b, c) respectively show the box plots of the FA, RD and AD values averaged over left and right fornix regions of interest. Black and blue boxes represent the values in the control and schizophrenia groups, respectively. Two, and three red asterisks respectively denote the p-value less than 0.05, 0.01.

**Figure 5:**
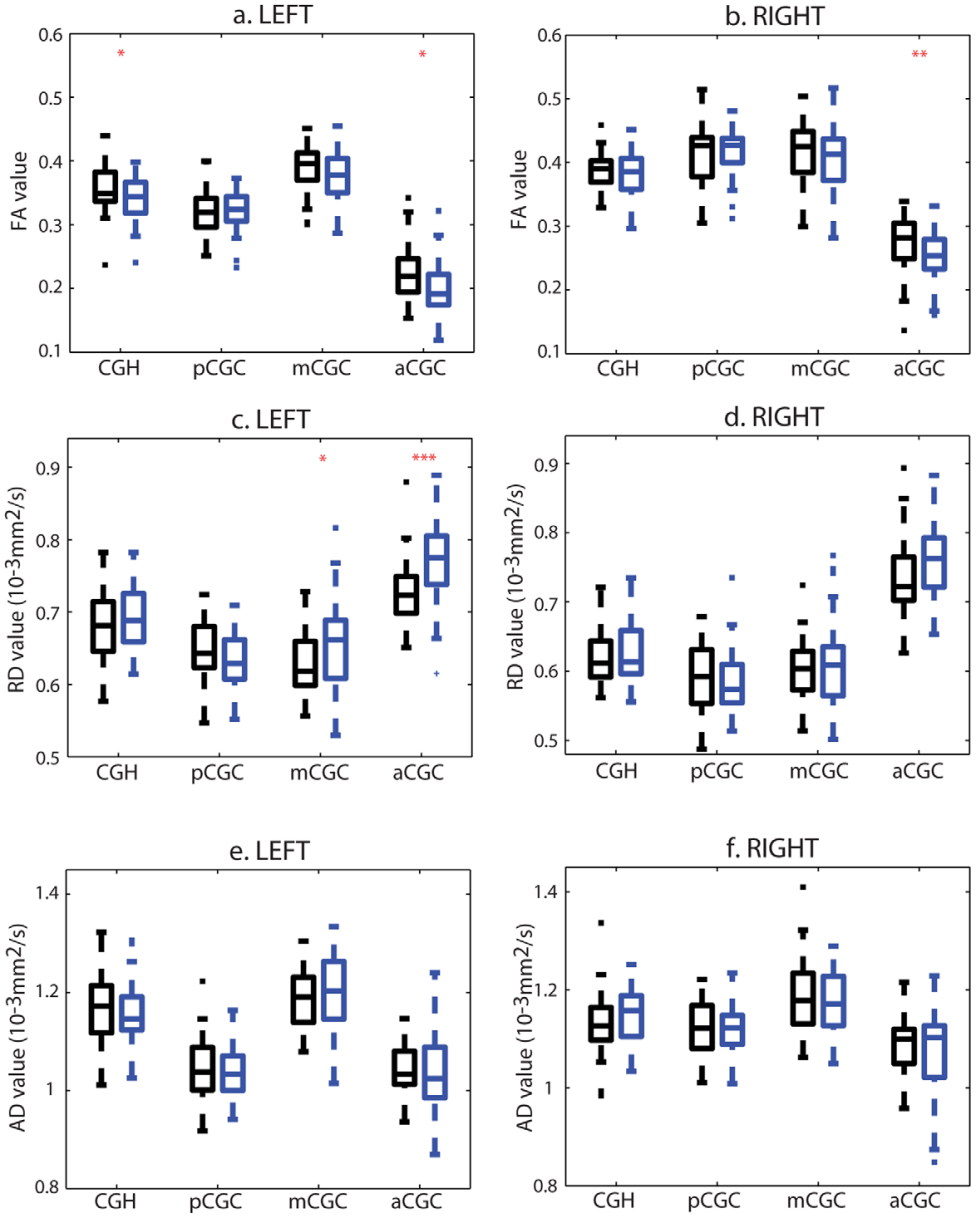
ROI-based analysis of the cingulum DTI measures. Panels (a, c, e) and (b, d, f) respectively show the box plots of the FA, RD and AD values averaged over left and right cingulum regions of interest. Black and blue boxes represent the values in the control and schizophrenia groups, respectively. One, two and three red asterisks respectively denote the p-value less than 0.1, 0.05 and 0.01. Abbreviations: CGH, cingulum adjoining the hippocampus; aCGC, anterior cingulum; mCGC, middle cingulum; pCGC, posterior cingulum.

**Figure 4** and **5** illustrate the box plots of the mean FA, mean RD and mean AD values in the fornix and cingulum bundles, respectively. After controlling for age, years of education, and illness duration, patients with schizophrenia had statistically significant FA reductions bilaterally in the fornix bundles (left: p<0.0005; right: p = 0.0492) compared to healthy controls. This was accompanied by increase in RD (left: p<0.0005; right: p = 0.0061) but not increase in AD of the fornix bundle (left: p = 0.9321; right: p = 0.8848). For the CGC bundle, patients with schizophrenia showed statistically significant RD increase in the left anterior CGC (p = 0.0041) with less significant decrease in FA bilaterally (left: p = 0.0563; right: p = 0.0487). No other group difference was found using ROI-based analysis.

### Tract-based Findings

**Figure 6:**
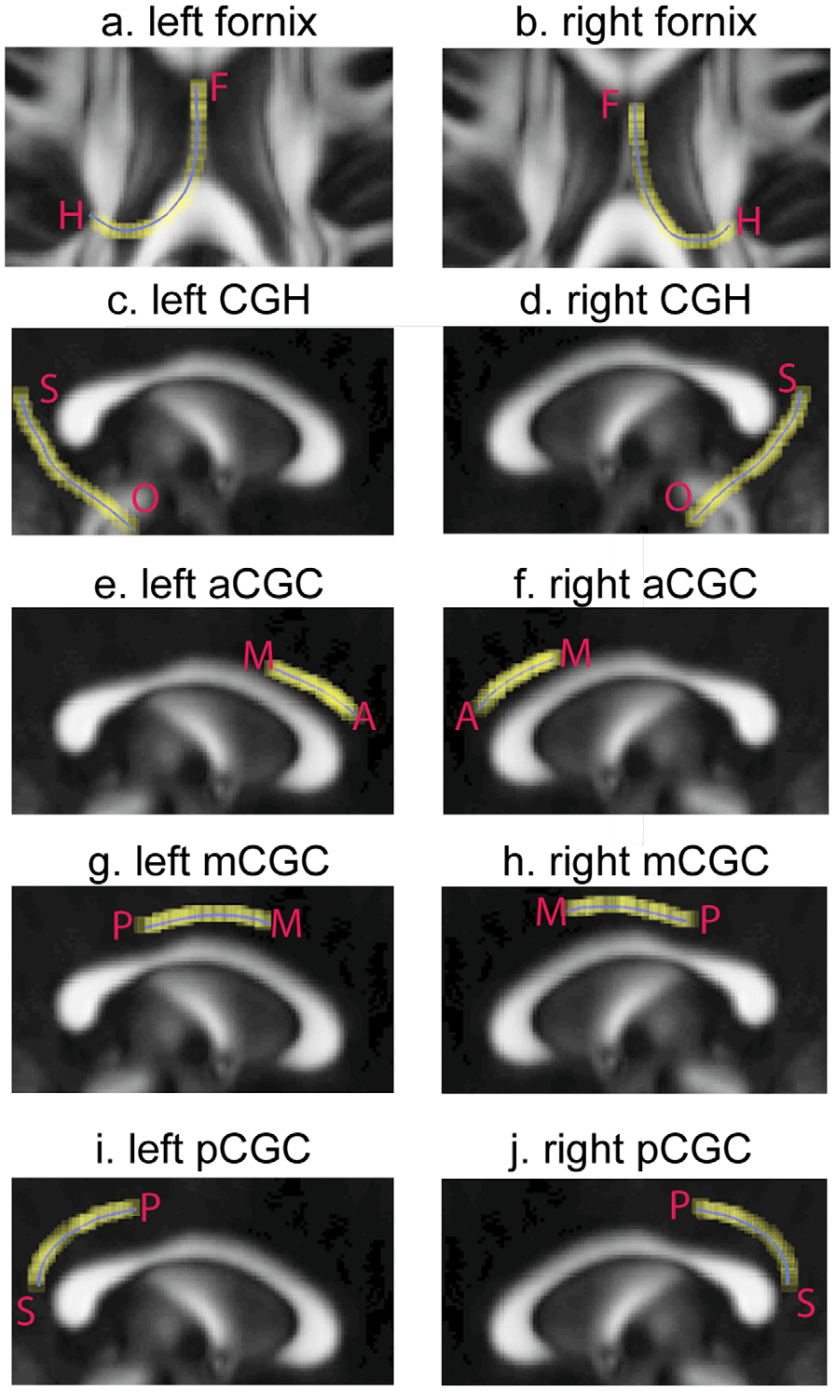
The fornix and cingulum fiber tracts (yellow lines) and their mean curves (blue line). Panels (a, b) respectively illustrate the left and right fornix, where “H” and “F” indicate the orientation of the fornix bundle from the hippocampus to the thalamus. Panels (c, d) respectively illustrate the left and right cingulum in the hippocampus (CGH), where “S” and “O” indicate the orientation of CGH from the splenium of the corpus callosum to the hippocampus. Panels (e–j) show the left and right cingulum in the cingulate (CGC) from the anterior, middle, to the posterior segments, where “A”, “M”, and “P” indicate the orientation of CGC from the anterior to the posterior.

**Figure 7:**
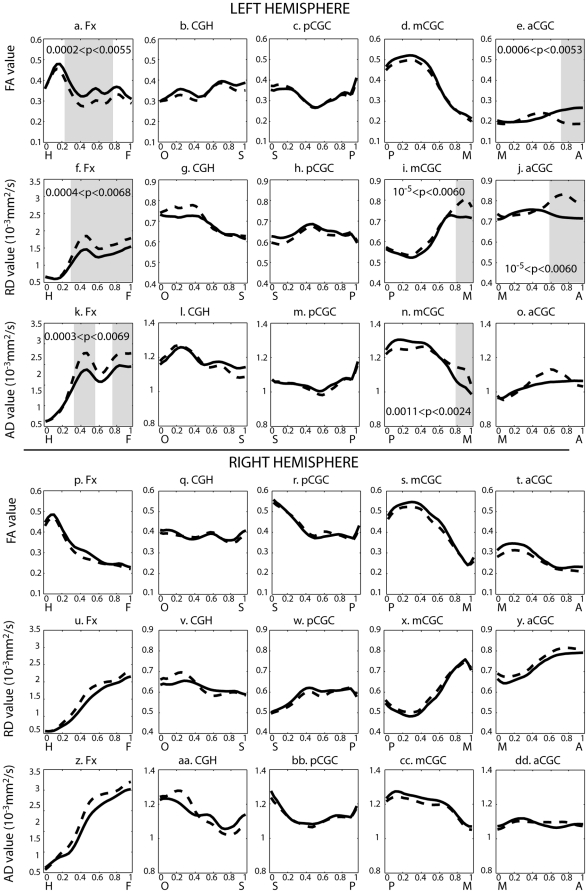
Tract-based analysis of DTI measures along fornix and cingulum. The top three rows respectively show FA, RD and AD profiles for left hemisphere, while the bottom rows show those for the right hemisphere. The panels from left to right respectively illustrate the profiles for regions of the fornix (Fx), cingulum adjoining the hippocampus (CGH), posterior cingulum (pCGC), middle cingulum (mCGC), and anterior cingulum tracts (aCGC). On each panel, solid line denotes the profile averaged over healthy controls, while dashed line denotes the profile averaged over patients with schizophrenia. The anatomical landmarks of each tract are given in **Figure 6**. Regions colored by gray are the anatomical locations with significant group difference at p<0.05 after Bonferroni correction. The range of p-values is indicated on each panel.

**Figure 6** illustrates the fornix and cingulum bundles and their mean tracts in the diffusion tensor images averaged over all the control subjects in this study. The FA, RD, and AD distributions along these mean curves are plotted in **Figure 7** with solid and dashed lines representing the averaged values in healthy controls and patients with schizophrenia respectively. Shaded rectangles correspond to regions with p<0.05 after Bonferroni correction.

Compared to control subjects, patients with schizophrenia showed reductions in FA along the specific regions of the left fornix (**Figure 7**(a)). It was also found that the RD and AD increased along middle region and superior region of the left fornix (**Figure 7**(f, k)). In the same location of the right fornix, patients with schizophrenia also showed a trend of the FA reduction and RD and AD increase although not at a significant level when compared with healthy controls (**Figure 7**(p, u, z)).

For the CGC bundle, the rostral region of the left anterior CGC showed lower FA and higher RD in patients with schizophrenia than in control subjects but no significant difference in AD (**Figure 7**(e,j,o)). In addition, patients with schizophrenia showed significant increases in both RD and AD but no significant FA reduction in the anterior segment of the left middle CGC (**Figure 7**(d,i,n)). No significant FA reduction or RD or AD increases were found in the left posterior CGC and CGH as well as the right CGC bundles.

### Relations with Clinical Measures

**Figure 8:**
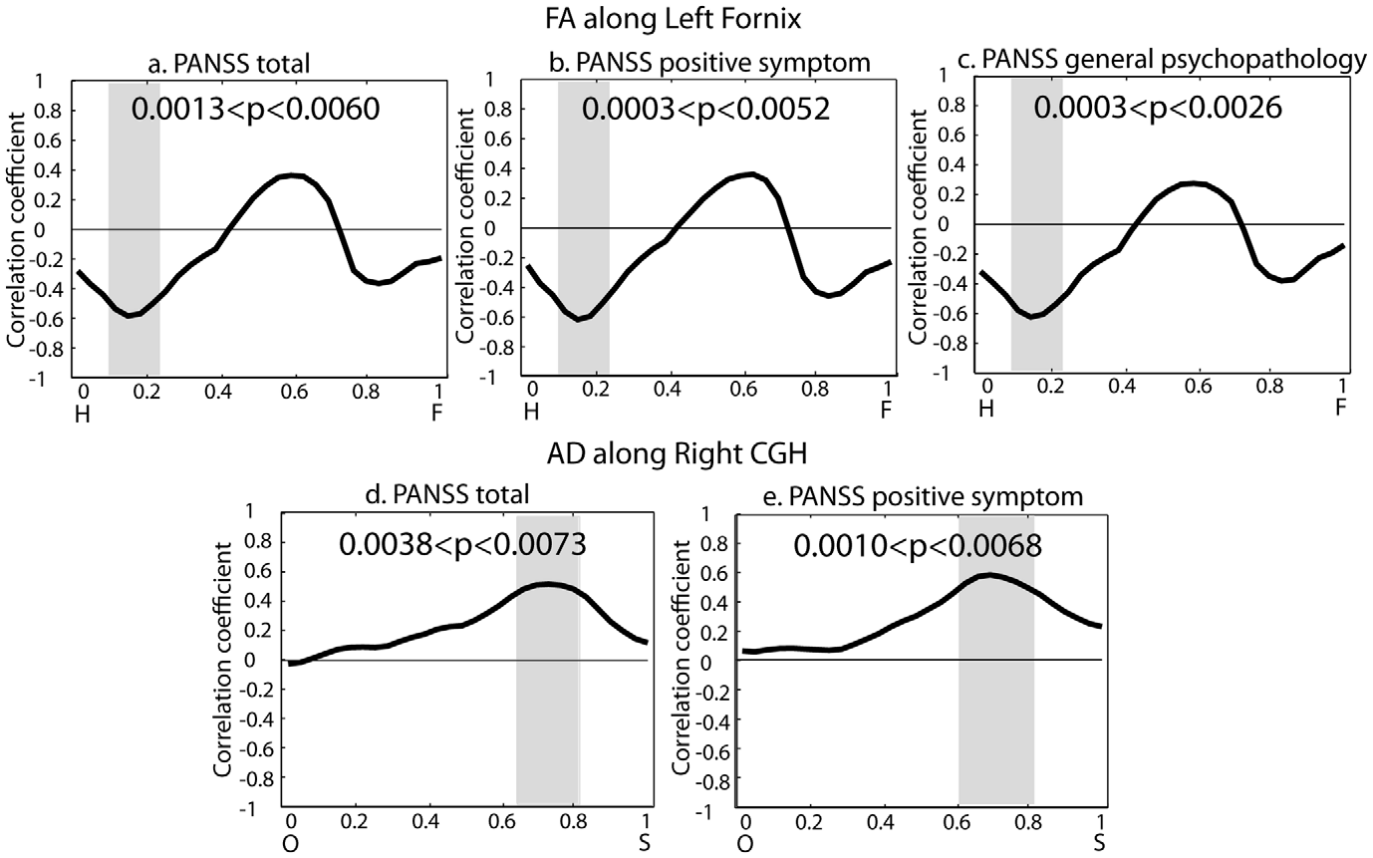
Partial correlation analysis between DTI measures and clinical scores in left fornix and right CGH. Panels (a–c) respectively show the correlation profile of FA values along the left fornix with PANSS total score, PANSS positive symptom score and PANSS general psychopathology score. Panels (d–e) show the correlation profile of AD values along the right CGH with PANSS total score and PANSS positive symptom score. The anatomical landmarks of each tract are given in **Figure 6**. Regions colored by gray are the anatomical locations with significant group difference at p<0.05 after Bonferroni correction. The range of p-values is indicated on each panel.

Even though the ROI-based analysis did not reveal significant correlations of the PANSS scores with any DTI measures, the tract-based analysis found significant negative correlations of the FA values in the loci of left fornix near to the hippocampus with PANSS total score (**Figure 8**(a)), PANSS positive symptom score (**Figure 8**(b)), and PANSS general psychopathology score (**Figure 8**(c)), indicating that a lower FA value correlated with greater psychopathology as shown by higher PANSS total, positive symptom, and general psychopathology scores. Additionally, significant positive correlations of the AD values with PANSS total score (**Figure 8**(d)) and PANSS positive symptom score (**Figure 8**(e)) were found at the loci of the right CGH near to the splenium, indicating that a larger AD value correlated with more severe symptomatology as shown by higher PANSS total and positive symptom scores.

## Discussion

This is the first study on schizophrenia to map regionally specific FA, RD, and AD differences along the entire fornix and cingulum in a detailed manner. In the traditional ROI-based analysis, we found significant reductions in FA and increases in RD in bilateral fornix, and RD increase in the left anterior cingulum in patients with schizophrenia compared to controls. Tract-based analysis further revealed specific loci of these white matter differences in schizophrenia in that FA reductions and RD increases occur in the region of the left fornix more distal from the hippocampus and in the rostral portion of the left anterior cingulum. In addition, the white matter properties at specific loci of the left fornix and the right cingulum were associated with psychopathology in patients with schizophrenia. These findings support precise disruptions of limbic-cortical integrity in schizophrenia.

Our finding of the mean FA reduction in the left fornix of patients with schizophrenia using tract-based analysis was consistent with findings of earlier studies where tract-based analysis were used [1], [25] but in contrast to studies using voxel-based analysis [42], [43]. Kuroki et al [44] specifically examined fornix abnormalities using a ROI approach and found reduction of FA within the fornix in schizophrenia, and together with data from tract-based studies, suggest that more precise definition and evaluation of the fornix are needed to better appreciate underlying white matter differences. In this study, more detailed examination of FA differences along the fornix added into the literature that FA reduction in schizophrenia occurred in the crux of the left fornix towards the midline where it merges with the crux of the right fornix and forms the body of fornix, which may be due to underlying demyelination, axonal damage or a combination of both processes as indicated by increases in RD and AD. Kumar et al. [45] speculated that the coincident increase of RD and AD is suggestive of reduction in axonal density because the consequent increase in axonal space results in less convergence of neural fiber tracts. Taken together, these evidences reinforced the theory of demyelination or caliber reduction in the crux of the left fornix in schizophrenia. While we observed significant reduction in the mean FA in the right fornix based on ROI analysis, there is only a trend of significance for FA decrease, RD and AD increase along the right fornix bundle using tract-based analysis.

Based on tract-based analysis, mean FA reduction in the left anterior CGC in schizophrenia is consistent with previous findings implicating the anterior cingulate region in schizophrenia [3], [21], [26]. This significant FA reduction in the rostral region of left anterior cingulum added to our understanding of cingulum pathology in terms of information about the precise location of white matter integrity changes in schizophrenia [3], [46]. Concurrently, the rostral region of the left anterior CGC also showed increased RD in schizophrenia, which together with FA reduction suggests that demyelination may underlie the white matter aberrations.

Regionally specific increase in RD was also found in the rostral region of the left middle CGC. There was a simultaneous increase in AD in this region, which was not picked up by the ROI-based analysis of the entire left middle CGC bundle. These results point to abnormalities in the bundle, which may be due to altered cytoarchitecture leading to increased CSF in this specific region. Compared to the anterior and posterior CGC, the middle CGC showed relatively higher FA and AD and lower RD. The relatively higher mean FA in the middle CGC compared to the anterior and posterior CGC may be related to the composition of long and short neural fibers and the changes along their connections with other brain regions [47]. It is likely that the mean curve representing the middle cingulum begins in a region with less fiber branches and hence, maintains higher FA before descending to both the anterior and posterior regions with more fiber branches to the other brain structures. It also results in AD decreasing and RD increasing simultaneously in the anterior and posterior CGC when compared to the middle CGC.

In our study, the white matter differences in the cingulum within schizophrenia were mainly limited to the anterior cingulum but not posterior cingulum, which indicates disruption of connections between the anterior cingulate and the prefrontal cortex which is germane to observed deficits of executive functioning in schizophrenia. Takei et al [21] found that white matter disruption in the cingulum was associated with increased reaction time in the Stroop task and Manoach et al [48] noted reductions in FA of the anterior cingulate gyrus to be associated with longer saccadic latencies in schizophrenia, underscoring the significance of the cingulum integrity in visuospatial attention and executing functioning in schizophrenia. In addition, the cingulum white matter differences in our study were more marked on the left cingulum, which was in line with most of earlier studies [2], [26], [49], [50]. This may indicate laterality effects which may be related to predominant right handedness of our study cohort and/or accentuation of left greater than right asymmetry involving white matter FA differences [3].

The negative correlations between FA along specific region of the left fornix and PANSS total, positive and general psychopathology scores are in contrast to the previous finding of absence of correlation between mean FA of fornix, delineated with the ROI-based approach, and psychotic symptomatology [51]. We did not find correlation between FA along the cingulum and PANSS scores which is in agreement with the findings from several [52], [53], [54] but not all earlier studies [55]. In view of the scarce findings, we admit that it is possible that severity of symptoms is tied to a pattern of white matter abnormalities that occur at specific loci along this fiber bundle instead of uniformly at one region across all subjects. At the same time, severity of schizophrenia symptoms may also be modulated by other regions as suggested by Mitelman et al [56]. Taken together, these findings suggest that the specific disruptions of white matter integrity along relevant neural tracts such as fornix and cingulum may exert a dominant effect, which could underlie psychotic psychopathology in schizophrenia. Several converging evidences from animal and human studies suggested the hippocampus as the brain origin of schizophrenia [57], [58]. Earlier neuroimaging studies revealed no cortical thinning [59] but anterior hippocampal deformity [60] in unaffected schizophrenic siblings, suggesting the hippocampal anatomy may confer vulnerability to the illness. Qiu et al. [58] examined the hippocampal morphology and its relation with the cortical thickness in the same cohort as that in this study and suggested the hippocampal-cortical disruptions in schizophrenia. Taken together with our results, the relation of schizophrenia diagnostic scores with the white matter disruption in the fornix near the hippocampus may suggest that brain tissue damage may be propagated due to the loss of white matter connections as schizophrenia symptoms become more severe. We notice that the correlation of FA with schizophrenia symptoms in the fornix is overlapped with the location of FA abnormalities in schizophrenia (Figure 7(a)) but not exactly the same. This may be due to the low degree of freedom since this correlation analysis was only performed in patients with schizophrenia.

There were several methodological strengths and limitations in this study. In terms of strengths, first, we employed the tractography on the averaged DTI such that the fornix and cingulum were delineated only in the subjects' overlapped region. Thus, it potentially reduced partial volume effects at the boundary of each structure on DTI measures. Second, we specifically examined the limbic white matter bundles using the DTI tractographic approach in addition to ROI-based analysis. This tractographic approach has higher specificity and sensitivity compared with conventional ROI or VBM approaches [22] in both the detection of abnormalities and the correlation of DTI measures with clinical scores. The extraction of the fornix and cingulum bundles is replicable based on Mori's DTI atlas and the delineation protocol as described above. Third, the combined investigations of white matter disruptions along both the fornix and the cingulum allows a more holistic understanding of the FA, RD, and AD differences within these limbic pathways traversing cortical and subcortical structures. Third, we analyzed the variation in FA, RD, and AD as a function of geometric position along the white matter bundle. Compared to the voxel-based analysis across the whole brain, our method is more specific and maps FA, RD, and AD differences along the entire fornix and cingulum. Our findings may provide guidance of the spatial location of resection in postmortem studies for understanding neuropathology of the fornix and cingulum in schizophrenia. In terms of limitations, our DTI data were not acquired in a cardiac gated fashion, which may cause motion induced by natural cardiac movement. As introduced in [61], the imaging acquisition could be further improved for better characterizing the small structures, such as the fornix. Furthermore, most of the patients with schizophrenia included in this study had been prescribed antipsychotic medications. However, earlier studies had not found medication related effects on FA [52], [62] and we did not find significant correlations between the antipsychotic dose and regional FA of these tracts. Future studies may want to better detail the disruptions of white matter integrity of fornix and cingulum in drug naïve individuals over illness course in order to tease out effects of psychotropic medications on the white matter differences prospectively.

In summary, we employed a tractographic approach to examine region specific FA, RD, and AD differences in the fornix and cingulum bundles. Our results suggested that regionally-specific white matter differences occurred at left fornix and the left anterior and middle cingulum in schizophrenia, which may be due to underlying demyelination of these fiber bundle regions, resulting in the disruption of limbic and cortical connectivities in schizophrenia. Furthermore, decreased FA at the left fornix and increased AD at right cingulum correlated with greater severity of psychotic symptoms in patients with schizophrenia, suggesting that disruption of white matter integrity may contribute towards the neural basis of clinical symptomatology. Considering the role of the hippocampus and cingulate gyrus in schizophrenia neurobiology, further investigation of region specific brain gray and white matter changes over time is needed to better understand the interactional nature of the relationship between these limbic structures and other related cortical and subcortical brain structures in the context of illness onset, progression and response to treatment.
